# Enhancing Nitrogen Removal in MBRs: From Theoretical Advances to Practical Applications

**DOI:** 10.3390/membranes16010013

**Published:** 2025-12-31

**Authors:** Jiayi Xun, Lu Wang, Fengwei Jia, Ziwen Han, Haoran Ma, Yiping Feng, Ying Zhao, Wenjuan Zhang, Dan Song, Jun Ma

**Affiliations:** 1 School of Marine Science and Technology, Harbin Institute of Technology at Weihai, Weihai 264209, China; 24s030043@stu.hit.edu.cn (J.X.); 23s030097@stu.hit.edu.cn (L.W.); 2022211478@stu.hit.edu.cn (F.J.); 23b929052@stu.hit.edu.cn (Z.H.); 24s030061@stu.hit.edu.cn (H.M.); 2023210559@stu.hit.edu.cn (Y.F.); yzhao16@hit.edu.cn (Y.Z.); 2 State Key Laboratory of Urban Water Resource and Environment, Harbin Institute of Technology, Harbin 150090, China; majun@hit.edu.cn; 3 Shandong Sino-European Membrane Technology Research Institute Co., Ltd., Weihai Key Laboratory of Water Treatment and Membrane Technology, Weihai 264209, China; 4 Tianjin Key Laboratory of Aquatic Science and Technology, School of Environmental and Municipal Engineering, Tianjin Chengjian University, Tianjin 300384, China

**Keywords:** membrane bioreactor, nitrogen removal mechanism, influencing factors, enhancing paths

## Abstract

Nitrogen, a prevalent water pollutant, is a major cause of eutrophication and the formation of black, odorous water bodies, posing significant threats to both ecological security and human health. Effectively controlling nitrogen pollution in wastewater is therefore essential for preserving aquatic ecosystems. The membrane bioreactor (MBR), which integrates the advantages of biological and membrane technologies, has attracted considerable attention for its application potential in wastewater nitrogen removal. This article elucidates the mechanisms and characteristics of nitrogen removal in MBR systems based on the latest research advancements. It provides an in-depth analysis of the key environmental factors affecting nitrogen removal efficiency and comprehensively summarizes enhanced processes centered on MBR technology. Furthermore, the article addresses corresponding strategies for mitigating MBR membrane fouling and offers suggestions and prospects for future research directions.

## 1. Introduction

The widespread occurrence of black and odorous water bodies, along with ecological crises such as eutrophication-driven algal blooms, has become a major obstacle to the sustainable use of global water resources [1,2]. As a major environmental pollutant, excessive nitrogen disrupts aquatic ecosystem balance and poses serious risks to human health. These risks arise through drinking water contamination and bioaccumulation in the food web, leading to conditions such as infant methemoglobinemia and increased susceptibility to chronic diseases [3,4,5]. Therefore, developing and applying advanced denitrification technologies for efficient nitrogen removal from wastewater has become a critical objective in environmental engineering. While conventional biological denitrification processes, such as activated sludge systems, enable basic nitrogen elimination, they are limited by large land footprints, high sludge production, and performance instability under variable water quality conditions. These constraints hinder compliance with increasingly stringent discharge standards and the growing need for water reuse [6,7,8].

**Figure 1:**
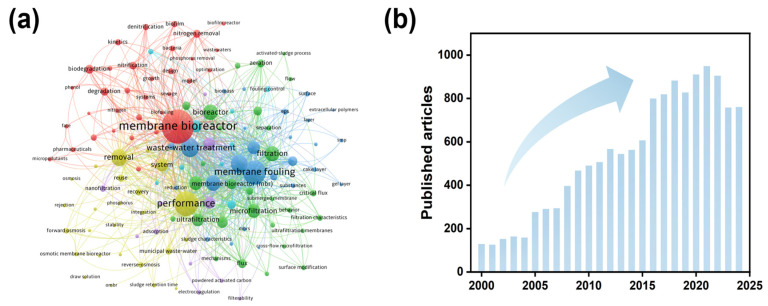
(**a**) Co-occurrence network of keywords related to MBR research. (**b**) The evolution of the number of scientific papers related to MBR. (2000–2025). Source: Web of Science Core Collection; Topic: “membrane bioreactor”; Searching date: October 2025.

MBR, which combine biological treatment with membrane filtration, show strong potential for advanced nitrogen removal due to their unique operational advantages [7]. The membrane modules can effectively retain activated sludge, allowing hydraulic retention time (HRT) to be decoupled from solids retention time (SRT) [8]. This creates a stable and enriched environment for slow-growing functional microorganisms, including nitrifying bacteria and anaerobic ammonium-oxidizing (anammox) bacteria. Moreover, the elevated biomass concentration in MBRs fosters a microenvironment with surface aeration and internal anoxia, a condition that facilitates simultaneous nitrification–denitrification (SND) and thereby enhances total nitrogen (TN) removal efficiency [9]. To date, MBR technology has been widely adopted in municipal and industrial wastewater treatment, with a global treatment capacity exceeding 20 billion liters per day [10,11]. From 2000 to 2025, research publications on MBR technology showed steady growth, reflecting both sustained academic interest and the promising role of MBRs in advanced denitrification, as illustrated in Figure 1.

Nevertheless, MBR systems continue to face technical challenges in achieving advanced nitrogen removal. Firstly, when treating complex industrial effluents containing high salinity, refractory organics, or toxic compounds, the metabolic activity of functional microorganisms is often inhibited. This can result in unstable nitrite accumulation, thereby impeding the implementation of efficient nitrogen removal pathways such as partial nitrification–denitrification coupled with anammox (PN/PD-AMX) [12,13]. Secondly, conventional MBRs generally rely on external carbon sources to drive denitrification, which increases operational costs and poses risks of secondary pollution. This issue is especially acute in low carbon-to-nitrogen (C/N) ratio wastewaters, where denitrification efficiency becomes markedly compromised [14,15,16]. Furthermore, membrane fouling not only elevates operational energy requirements and maintenance expenses but may also disrupt microbial community structure due to frequent cleaning, thereby undermining process stability [17,18]. Although novel microorganisms such as denitrifying anaerobic methane-oxidizing (DAMO) bacteria enable synergistic nitrogen and methane removal without nitrous oxide (N_2_O) emissions [19], their slow growth kinetics and inefficient substrate mass transfer often lead to start-up periods exceeding one year, making them currently impractical for full-scale implementation.

To address these challenges, researchers worldwide have pursued multi-faceted innovations to enhance nitrogen removal in MBR systems. From a mechanistic perspective, strategies such as optimizing nitrite accumulation via control of dissolved oxygen (DO) and SRT [20,21], or developing membrane-aerated membrane bioreactors (MAMBR) to promote the enrichment of DAMO bacteria and anaerobic ammonium-oxidizing bacteria (AnAOB), have established a microbiological basis for more efficient nitrogen removal pathways [22]. At the process level, integrating MBR with biological systems such as anoxic/oxic (A/O) [23] or anaerobic/anoxic/oxic (A/A/O) [24], or combining it with pretreatment methods including coagulation [25], ozonation [15], and powdered activated carbon adsorption [26], has been shown to improve the elimination of refractory organics, alleviate membrane fouling, and enhance the metabolic activity of functional microorganisms [27]. Further improvements have been achieved through the regulation of sludge and biofilm environments by incorporating suspended carriers [28,29], sulfur-based media [26], or “carrier–carbon source” hybrid systems, which create tailored microhabitats for specific functional microbes and thereby facilitate targeted processes such as autotrophic denitrification and anaerobic ammonium oxidation.

Based on this foundation, this review systematically analyzes the core mechanisms of biological nitrogen removal in MBR systems, focusing particularly on the impact of key operational parameters—including pH, temperature, DO, C/N ratio, HRT/SRT, aeration strategy, and flow distribution—on denitrification performance. It also outlines the classification and current application landscape of MBR configurations. Furthermore, the study synthesizes enhancement strategies for advanced nitrogen removal in MBR systems across four dimensions: hybrid biological processes, integrated pretreatment, sludge modification, and biofilm regulation. Finally, considering existing technical challenges, prospective research directions and engineering application recommendations are proposed, aiming to provide theoretical insights and practical guidance for the optimization and upgrading of MBR-based advanced nitrogen removal technologies.

## 2. Mechanism of Biological Denitrification in MBR Systems and Key Influencing Factors

### 2.1. Biological Denitrification Mechanism

**Figure 2:**
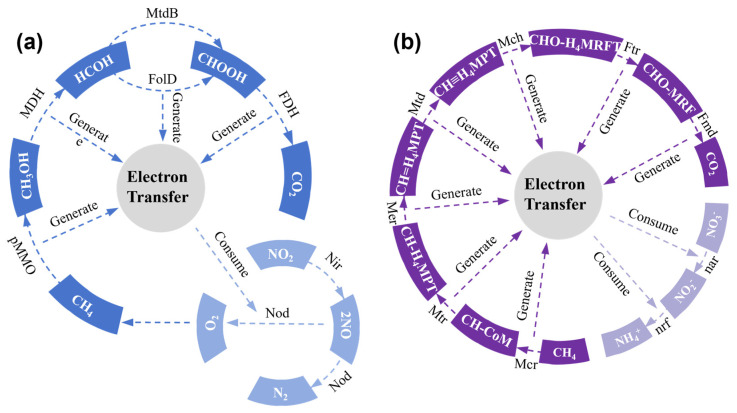
(**a**) NO_2_^−^ driven and (**b**) NO_3_^−^ driven methane anaerobic oxidation processes.

The membranes in MBR systems retain microbial biomass, preventing sludge washout and enabling the enrichment of slow-growing nitrifying bacteria [30]. This retention maintains high biomass concentration and metabolic activity, establishing favorable conditions for efficient nitrogen removal [31,32]. Furthermore, the elevated sludge concentration promotes the formation of oxygen-gradient microenvironments within flocs—aerobic at the surface and anoxic/anaerobic in the interior—thereby facilitating simultaneous nitrification and denitrification. Regarding nitrogen removal pathways in MBRs, most studies focus on single-stage or two-stage configurations employing conventional nitrification–denitrification. Reported TN removal rates ranged from 46 to 60% in single-stage aerobic MBRs and 60–80% in two-stage nitrification–denitrification MBRs. Intermittently aerated MBRs achieve over 80% TN removal with a substantially lower energy demand, indicating that operational modifications can enhance efficiency and reduce the necessary energy input [33]. Emerging pathways such as anammox and autotrophic denitrification offer promising alternatives for improving MBR performance [34,35,36] Nitrite (NO_2_^−^) accumulation plays a central role in governing these pathways, serving both as an intermediate in partial denitrification (PD) and as the key substrate in the anammox process (AMX) [37]. Thus, achieving stable NO_2_^−^ accumulation via partial nitritation anammox (PN/AMX) has become a major research direction [38]. The primary process involves the oxidation of ammonia nitrogen (NH_3_) to NO_2_^−^ by ammonium-oxidizing bacteria (AOB), coupled with the reduction in nitrate (NO_3_^−^) to NO_2_^−^ due to oxygen (O_2_) consumption in the water body. In this context, residual NH_3_ acts as an electron donor and the generated NO_2_^−^ serves as an electron acceptor, enabling the simultaneous removal of NH_4_^+^ and NO_2_^−^ via anaerobic ammonium oxidation (anammox), resulting in the production of nitrogen gas (N_2_) [39], as illustrated in Figure 2a. In this cycle, methane (CH_4_) is activated by particulate Methane Monooxygenase (pMMO) to methanol (CH_3_OH), which is further oxidized to formaldehyde (HCOH) by Methanol Dehydrogenase (MDH) and to formate (CHOOH) by Formate Dehydrogenase (FDH). These intermediates generate electrons through pathways involving FolD, which are transferred to drive the reduction in NO_2_^−^: NO_2_^−^ is first converted to nitric oxide (NO) by Nitrite Reductase (Nod), and then NO is reduced to nitrogen gas (N_2_) by Nod, while oxygen (O_2_) is also involved in related reactions, with carbon dioxide (CO_2_) as a by-product of complete methane oxidation.

Long-term studies show that free ammonia [40] and sulfides [41] can selectively inhibit nitrite-oxidizing bacteria (NOB). Moreover, due to the higher oxygen affinity of NOB compared to AOB, DO control and SRT regulation can be used to modulate the relative abundance and activity of AOB and NOB, thereby promoting nitrite accumulation [42,43]. However, the instability of the PN process has hindered the large-scale application of PN/PD-AMX systems [44,45]. Ma et al. [46,47] successfully enriched anammox bacteria by forming anammox biofilms or anammox granular sludge, enabling rapid initiation of the anammox process. Zhang et al. [48] clarified the metabolic pathways of denitrifying microorganisms and their environmental selection criteria, developing a method to enrich denitrifying functional bacteria (DNB) for stable NO_2_^−^ accumulation. Furthermore, the discovery of DAMO microorganisms has introduced a novel denitrification pathway that utilizes methane as a potential electron donor for PD, as illustrated in Figure 2b. In this cycle, CH_4_ is involved in reactions with methyl-coenzyme M reductase (Mcr) and a series of coenzymes (e.g., CH≡H_4_MPT, CH_2_=H_4_MPT, CH_3_-H_4_MPT, CH_3_-CoM) and enzymes (e.g., Methane Methyltransferase (Mtr), Methyl Reductase (Mer), Methyl-coenzyme M Reductase-associated Protein (Mdr), Methane Coenzyme Hydrogenase (Mch), Fe-hydrogenase (Frh)), generating electrons that drive the reduction in NO_3_^−^ and NO_2_^−^: NO_3_^−^ is first reduced to NO_2_^−^ by Nitrate Reductase (nar), and NO_2_^−^ is then reduced to NO and N_2_ by Nitric Oxide Reductase (nrf), with CO_2_ as a by-product. In the nitrate-type metabolism driven by DAMO, nitrate serves as the terminal electron acceptor, activating Mcr and ultimately reducing CH_4_ to CO_2_ while converting nitrate to nitrite. In the nitrate-type metabolism driven by DAMO, nitrate serves as the terminal electron acceptor, activating methyl-coenzyme Mcr and ultimately reducing CH_4_ to CO_2_ while converting nitrate to nitrite. In the nitrite-type metabolism, DAMO reduces nitrite to NO, which is subsequently converted to N_2_, while simultaneously driving aerobic methane oxidation via methanesulfonate reductase [49]. Although the DAMO-coupled PN/AMX process enables simultaneous nitrogen removal and methane oxidation without releasing the greenhouse gas N_2_O, the slow growth of DAMO organisms—largely due to inefficient methane mass transfer—typically results in start-up periods exceeding one year [50].

To address this challenge, Nie et al. [51] developed a MAMBR that enriches functional microorganisms using high-concentration mixed sludge and shortens the start-up period of DAMO processes. In a complementary approach, Xue et al. [52] demonstrated that incorporating iron-modified granular activated carbon into activated sludge promotes the conversion of NH_3_ and NO_2_^−^, while simultaneously enhancing the relative abundance of DAMO archaea and anammox bacteria. These findings indicate that increasing the biomass and metabolic activity of key functional microorganisms (e.g., AOB, DAMO, anammox bacteria) offers a practical pathway for upgrading MBR systems and achieving advanced nitrogen removal. These microorganisms exhibit high substrate affinity, strong environmental resilience, and low greenhouse gas emissions [53,54,55].

### 2.2. Factors Influencing Biological Denitrification

The activity of denitrifying microorganisms is influenced not only by biological metabolic pathways but also by external conditions. Variations in microbial activity under different conditions can affect the actual denitrification performance of MBR systems [56]. This analysis examines the impact of pH, temperature, DO, C/N ratio, HRT, SRT, aeration methods, and water distribution patterns on biological denitrification efficiency.

#### 2.2.1. pH, Temperature, and Dissolved Oxygen

The efficiency of biological denitrification in MBR systems is governed by a complex interplay of key environmental parameters, primarily pH, temperature, and DO, which collectively regulate microbial metabolism, community structure, and process kinetics.

pH serves as a master variable, critically influencing microbial physiology, enzyme activity, and substrate bioavailability [57,58]. It directly affects the metabolic activity and competitive fitness of functional microorganisms by altering cell membrane permeability and the chemical speciation of nitrogen substrate. Simultaneously, pH-induced changes in microbial surface charge can modify community interactions and floc stability. A pH range of 7–8 is generally optimal for denitrifying communities [59], supporting high activity for AOB, DNB, DAMO microorganisms, and Anammox bacteria, the latter having a reported optimum between 6.7 and 8.3 [60]. Deviations from this range can inhibit key enzymes and disrupt microbial aggregation.

Temperature modulates reaction rates and microbial growth kinetics, thereby indirectly shaping denitrification performance [57]. Elevated temperatures, typically around 30–35 °C [61], often favor processes like partial nitrification and anammox by selectively enhancing the growth rate of AOB over NOB [58]. However, temperature fluctuations in practical systems can lead to instability in microbial activity and treatment efficiency.

DO concentration is a pivotal factor for controlling the balance between nitrification and denitrification. While nitrification requires oxygen, low DO levels (e.g., 0.5–1.5 mg/L) are conducive to denitrification. Operating at a DO around 1 mg/L can create SND conditions, where oxygen is depleted in the outer layers of sludge flocs, allowing anoxic denitrification to proceed in the inner zones [62]. This strategy offers a marked enhancement in total nitrogen removal without compromising ammonia oxidation, paving the way for nitrogen removal that is both low-energy and highly efficient [63]. A summary of the optimal ranges and primary effects of these and other critical factors is provided in Table 1.

#### 2.2.2. Carbon-to-Nitrogen Ratio

The C/N ratio in aquatic systems is also a critical factor influencing the denitrification capacity of functional microorganisms [9]. Traditional biological denitrification theory holds that 2.86 g of carbon source are required to reduce 1 g of NO_3_^−^ to N_2_. However, in practice, the actual amount often exceeds this theoretical value. In general, when the C/N ratio in a water body falls below 5, the available carbon source becomes insufficient to initiate denitrification, leading to poor denitrification efficiency [67]. To overcome carbon source dependency, numerous researchers had continuously refined processes to achieve more efficient denitrification pathways. In a study by Gao et al. [68] utilizing a PN/AMX coupled A/A/O process for municipal wastewater treatment, an operational C/N ratio of 3.4 led to a shift in microbial community: the abundance of *Nitrosomonas* increased from 0.32% to 1.51%, while that of *Nitrosococcus* decreased from 1.25% to 0.11%. Furthermore, anammox bacteria on carriers showed a 2.45% higher relative abundance compared to those in flocs, further evidencing the system’s excellent denitrification performance. Similarly, Choi et al. [64] demonstrated that increasing the carbon-to-nitrogen ratio from 1.5 to 3, along with controlled inoculation of anammox bacteria, could selectively inhibit *Nitrosomonas* and stabilize the PN/A process. This operational strategy enhanced the denitrification efficiency from 63.4 ± 1.5% to 81.4 ± 4.2%.

#### 2.2.3. HRT and SRT

An excessively prolonged HRT can induce nutrient depletion, slowing microbial metabolism and even triggering endogenous respiration [66]. Declining microbial activity impairs sludge function and reduces effluent quality. Conversely, a short HRT may wash out functional microorganisms, impeding microbial enrichment and reducing sludge concentration [69]. In a pilot-scale study, Sohn et al. observed that as HRT decreased from 7 day to 1 day, AOB outcompeted NOB, leading to pronounced nitrite accumulation and a sharp decline in nitrification efficiency from 40% to 11%. This was accompanied by a significant reduction in microbial diversity and a simplification of functional community structure, underscoring the critical role of appropriate HRT control in sustaining microbial activity and treatment performance [70]. Chen et al. [71] through experiments with MBR under varying conditions, identified an optimal HRT of 10 h. HRT also influences microbial c8ommunity structure, with reactor performance optimized when HRT approximates the generation time of key microorganisms. Additionally, HRT affects hydraulic shear stress, which directly shapes biofilm structure and the morphology of sludge particles.

Modifying the solids retention time (SRT) governs denitrification performance and has a pronounced effect on process stability. SRT governs mixed liquor properties such as viscosity, biomass concentration, microbial community composition, particle size distribution, and cell surface characteristics. Typically, an SRT of 20–50 days supports complete nitrification and denitrification while minimizing sludge disposal frequency [72]. However, a long SRT can lead to sludge aging, disintegration of flocs, and cell lysis, thereby impairing nitrification/denitrification kinetics and reducing overall denitrification performance. In contrast, a short SRT prevents the effective retention and proliferation of slow-growing nitrifying bacteria, resulting in incomplete nitrification and lower denitrification efficiency. Furthermore, changes in SRT directly influence the growth dynamics of activated sludge [73]. Huang et al. [74]. reported that both sludge concentration and volatile biomass increase with rising SRT within reactor systems.

#### 2.2.4. Aeration and Water Distribution Methods

The aeration method directly governs the distribution and concentration of DO in different process zones, thereby influencing microbial enrichment and the selection of denitrification pathways [75]. Conventional aeration methods require high aeration rates and considerable energy, yet their treatment efficacy fails to meet new water quality requirements [76]. Rational control of aeration volume and DO concentration is pivotal for achieving low-energy deep denitrification. Tang et al. [77] developed a micro-gradient aeration apparatus where multi-stage low-DO zones facilitated simultaneous partial nitrification and denitrification, yielding satisfactory denitrification efficiency (93% ± 3%).

Organic loading exerts a significant influence on microbial growth and succession. Consequently, modifying the water distribution pattern—specifically, shifting from single-source to multi-stage inflow—alters the spatial distribution of organic matter across the treatment process, thereby directly impacting denitrification efficiency. In a study by Choi et al. [65], a laboratory-scale reactor with a three-stage influent distribution (40%, 30%, and 30%) was constructed. It was found that the multi-stage anaerobic configuration enabled organic matter to aid in NOB suppression, thereby enhancing the anammox contribution and achieving a nitrogen removal efficiency of 86.1% ± 3.7%.

The denitrification performance of MBR systems is governed by a suite of interacting parameters—pH, temperature, DO, C/N ratio, HRT, SRT, aeration method, and water distribution pattern. The effectiveness of integrated processes capitalizes on this principle, as they are designed to coordinately regulate these factors, thereby enhancing MBR performance and enabling deep denitrification.

## 3. Principles and Applications of Membrane Bioreactors

### 3.1. Pollution Removal Mechanisms in MBR Systems

The conventional MBR process is a technology that integrates membrane separation with biological treatment [65]. It primarily employs microbial enrichment to enhance the degradation efficiency of organic pollutants in water bodies; membrane filtration controls the high activated sludge concentration within the reactor while simultaneously reducing sludge discharge volumes by maintaining a low sludge loading rate [18]. Within MBR systems, the mixed liquor suspended solids (MLSS) concentration can reach 8000–10,000 mg/L or higher, while the SRT can be extended to over 30 days [78] Furthermore, the MBR’s thorough retention of macromolecular organic matter and microbial communities ensures effective temporal and spatial contact between them, thereby promoting the growth of functionally significant microorganisms with longer generation times. The extensive proliferation of functional microorganisms—such as nitrifying (e.g., AOB, NOB), denitrifying, and polyphosphate-accumulating organisms—within the system [79,80,81,82] enables advanced nutrient removal, leading to the deep purification of wastewater.

### 3.2. Classification and Application of MBR

MBR systems can be classified according to several criteria. Based on membrane module configuration and installation, they are categorized as combined MBR (CMBR). According to aeration requirements, they are divided into aerobic MBR (AMBR) and anaerobic MBR (AnMBR). Regarding pressure operation modes, the categories include external pressure recirculating MBR (EMBR) and submerged suction MBR (SMBR). Furthermore, membrane modules themselves can be classified by their configuration and material into tubular MBR, flat-sheet MBR, hollow-fiber MBR, MBR with organic membranes, and MBR with inorganic membranes, as summarized in Table 2.

**Figure 3:**
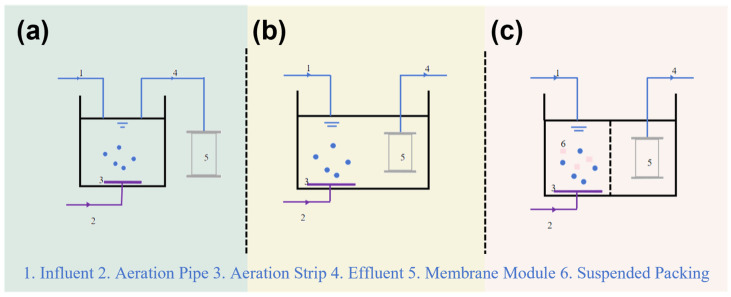
(**a**) Recirculated MBR; (**b**) Submerged MBR; (**c**) Combined MBR.

Currently, EMBR, SMBR and CMBR represent the most widely adopted MBR configurations. In a RMBR, also referred to as cross-flow MBR, membrane modules are positioned externally to the bioreactor and connected via piping and pumps. Pressure is applied to drive the mixed liquor through the membrane for solid–liquid separation, with the concentrated stream recirculated back to the bioreactor [83] EMBR primarily utilizes tubular or flat-sheet membrane modules, and its process flow is depicted in Figure 3a. Since the membrane modules and bioreactor are housed separately, mutual interference is minimized, allowing for easier regulation, control, cleaning, and replacement. However, the reliance on recirculation and pressurization pumps leads to a relatively high energy demand. In addition, strong hydraulic shear forces can disrupt microbial communities and compromise effluent quality.

In a SMBR, membrane modules are directly immersed in the bioreactor, where microorganisms degrade organic pollutants in the aeration tank. The permeate is extracted by gravity, suction pumps, or vacuum-induced negative pressure [84,85]. SMBR mainly employs hollow fiber membrane modules, with a typical process flow shown in Figure 3b [86,87]. Compared to EMBR, SMBR reduces energy use by approximately 90%. Moreover, reduced shear stress on microbial flocs promotes biological activity and fosters more stable effluent quality [84]. A key drawback, however, is the faster rate of membrane fouling, and the cleaning or replacement of modules is more cumbersome, generally resulting in lower permeate flux than EMBR [88]. CMBR is a subtype of SMBR. Its main distinguishing feature is the incorporation of functional packing materials into the bioreactor to address specific operational needs [89]. A schematic diagram of its process flow is provided in Figure 3c.

**Figure 4:**
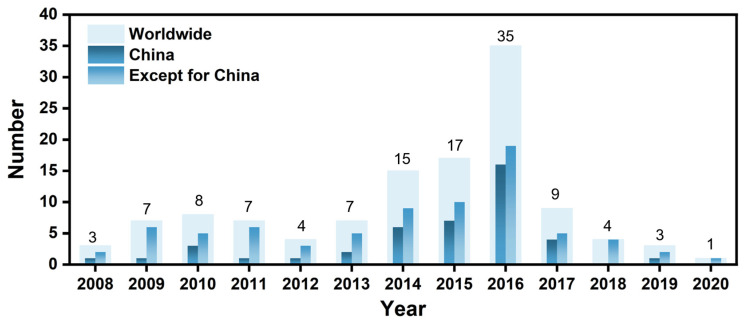
The number of large-scale (more than 100,000 tons) MBR water plants built worldwide from 2008 to 2020.

Due to its advantages, MBR global application has expanded steadily since the early 21st century, with facilities increasing in scale [90,91]. As shown in Figure 4, since 2008, dozens of new MBR facilities have been commissioned annually for both municipal and industrial wastewater treatment. According to data from the international MBR network, since the first 100 million liters per day (MLD) MBR plant was commissioned in 2007, more than 30 MBR facilities with capacities exceeding 100 MLD had been installed worldwide by 2016, most of which are located in China. By 2019, this number had doubled. The total treatment capacity of MBR systems worldwide now exceeds 20 billion liters per day (GLD). Given that global annual wastewater generation is estimated at 1000 GLD, MBR technology accounts for about one-twentieth of the world’s total treatment capacity.

Although MBR application research started relatively late in China, its development has been rapid. Several large-scale MBR systems with daily treatment capacities exceeding 10,000 tons are now in operation, including the Guangzhou Jingxi Wastewater Treatment Plant (10 × 10^4^ m^3^/d), Nanjing Chengdong Wastewater Treatment Plant (15 × 10^4^ m^3^/d), Kunming Tenth Wastewater Treatment Plant (15 × 10^4^ m^3^/d), and Wuhan Sanyintan Wastewater Treatment Plant (20 × 10^4^ m^3^/d).

### 3.3. Emerging MBR Configurations

Building upon conventional classifications, recent research has focused on hybrid systems that integrate novel principles to enhance functionality, particularly for energy efficiency and resource recovery. Two notable emerging configurations are the hybrid electrochemical MBR (e-MBR) and the bioelectrochemical-assisted anaerobic MBR [92].

e-MBR incorporates electrodes directly into the bioreactor. Applying a small electric field or current induces several beneficial effects: (1) in situ generation of oxidants (e.g., hydrogen peroxide, reactive oxygen species) at the anode that can degrade refractory organic compounds and mitigate membrane fouling; (2) electrophoresis and electrocoagulation that alter sludge properties, improving settleability and reducing fouling propensity; and (3) potential direct stimulation of microbial activity. This integration offers a promising alternative to chemical cleaning and pretreatment, though its scalability is influenced by electrode material costs and operational energy consumption [93,94]. For example, Niu et al. [95] constructed an electrochemical anaerobic membrane bioreactor (EC-AnMBR), which achieved high pollutant removal efficiency and a 107.6% increase in methane yield under optimal voltage, and used machine learning to identify key factors affecting methane productivity and membrane fouling. Zhang et al. [96] developed the electrochemical resource-recovery anaerobic membrane bioreactor (eRAnMBR) with dual anodes, realizing 95% membrane fouling mitigation, 93.97% methane purity, and efficient recovery of nitrogen, phosphorus as struvite.

The bioelectrochemical-assisted anaerobic MBR synergistically combines an AnMBR with a microbial electrochemical system (BES) [97]. In this configuration, electroactive bacteria in the anode biofilm oxidize organic matter, releasing electrons and protons. These electrons are then transferred to the cathode, where they can drive reduction reactions. This setup enables simultaneous wastewater treatment, energy recovery and enhanced removal of nutrients and recalcitrant pollutants. The bioelectrochemical reactions can create unique redox conditions that favor specific metabolic pathways, such as autotrophic denitrification, thereby achieving advanced nitrogen removal alongside energy generation [98].

These advanced configurations exemplify the ongoing evolution of MBR technology beyond solid–liquid separation, moving towards multifunctional systems capable of energy-positive operation and enhanced resource recovery [92].

## 4. Enhanced MBR Deep Denitrification Process

### 4.1. Enhanced Denitrification via Coupled Bioprocesses

To improve nitrogen removal, enhance effluent quality, reduce fouling, lower costs, researchers have explored integrating complementary processes with MBR to strengthen overall nitrogen removal performance. As summarized in Table 3, the choice of coupled processes largely depends on wastewater characteristics and other operational constraints [99]. Thanks to its high contaminant retention capacity, MBR is generally placed at the terminal stage of combined systems to ensure the biological safety of the effluent. The main role of front-end coupled processes is to improve biodegradation and support synergistic denitrification [100]. These integrated systems can achieve nitrogen removal efficiencies above 72.3% [9].

In the multi-stage A/O-MBR process, sequential A/O units creates a gradient degradation environment, enhancing microbial pollutant degradation capacity [102]. At the same time, the membrane modules enhance microbial retention, minimizing the loss of functional microbial communities. This dual effect improves nitrogenous pollutant removal, achieving efficiencies of over 78.6% [109]. Mechanistically, this configuration provides multiple redox zones, favoring diverse microbial communities. While it offers robust performance under fluctuating loads, the increased number of reactors elevates capital costs and footprint, potentially limiting its scalability for space-constrained sites. Additionally, the improvement of sludge properties in multi-stage reactors helps mitigate membrane fouling and lower operational costs [110].

The A/A/O process, widely used for municipal wastewater, effectively removes nitrogen and phosphorus simultaneously. Its integration with MBR leverages the membrane’s ability to efficiently retain polyphosphate-accumulating organisms and nitrifying bacteria, increasing denitrification efficiency to 78.6% ± 6.5% [104,105]. The key distinction from the A/O-MBR lies in its dedicated anaerobic zone for enhanced biological phosphorus removal, making it ideal for nutrient-rich streams, though it demands more precise control of recirculation flows and sludge retention times [106]. Moreover, by avoiding the need for multiple A/O units in series, this configuration significantly improves process compactness and can serve as a competitive alternative to more complex multi-stage A/O-MBR systems [111]. The anaerobic/anoxic/oxic/anaerobic-membrane bioreactor (A/A/O/A-MBR) configuration introduces an additional anoxic zone, which expands denitrification sites and improves simultaneous nitrification and denitrification [112]. This modification raises the total nitrogen removal efficiency to over 88.4% ± 7.6%, offering a feasible and relatively cost-effective strategy for upgrading existing A/A/O-MBR facilities with moderate retrofitting costs and minimal energy penalty [24].

**Figure 5:**
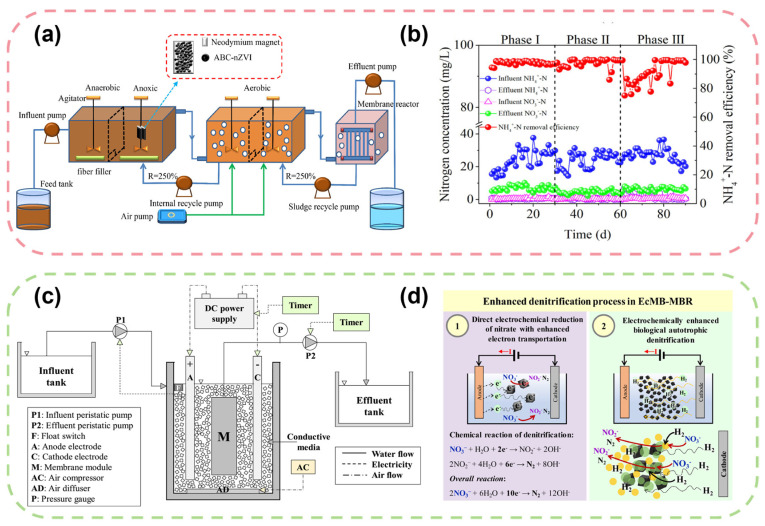
Schematic diagrams and performance curves of enhanced denitrification processes based on coupled bioprocesses and pretreatment. (**a**) Schematic diagram of the A/A/O-MBR process modified by ABC-nZVI; (**b**) Variations in nitrogen concentrations and NH_4_^+^-N removal efficiency during the operation of ABC-nZVI modified A/A/O-MBR [109]; (**c**) Schematic diagram of the electroconductive moving bed membrane bioreactor (ECMB-MBR) system; (**d**) Enhanced denitrification mechanism and related chemical reactions in ECMB-MBR [128].

Particularly noteworthy is that enhancing denitrification by adding functional materials to the A/A/O-MBR system has become an important technical direction. As shown in Figure 5a,b, Wang et al. demonstrated that when treating low C/N municipal wastewater, the addition of algal biochar-supported nano zero-valent iron (ABC-nZVI) to the anoxic zone of an A/A/O-MBR system extended the duration of high-efficiency denitrification to 9 h, significantly improved total nitrogen removal capacity, and ensured that the effluent NH_4_^+^-N met national discharge standards [109]. This material not only serves as an electron donor to promote autotrophic denitrification but also further enhances the system’s nitrogen removal performance by improving the biodiversity of activated sludge and enriching functional genes related to denitrification. While the material cost of additives like ABC-nZVI presents an economic consideration, their long-term benefits in reducing external carbon source consumption and stabilizing performance under low C/N conditions can improve overall cost-effectiveness. The scalability of this approach depends on the sustainable production and dosing management of these functional materials.

For wastewater containing antibiotics and organic compounds, the upflow anaerobic sludge blanket-anoxic/oxic-membrane bioreactor (UASB-A/O-MBR) process shows distinctive benefits. The UASB acts as a pretreatment step, degrading a portion of organic pollutants and reducing antibiotic toxicity via anaerobic digestion. This alleviates inhibitory effects on functional microorganisms in the subsequent A/O stage, thereby improving biodegradation and denitrification efficiency. This hybrid system demonstrates good scalability for high-strength industrial wastewater treatment, as the UASB stage significantly reduces the organic load to the downstream MBR, lowering aeration energy requirements and membrane fouling propensity. The methane produced in the UASB can also be recovered, partially offsetting operational energy costs, thereby improving the overall energy balance and economic viability of the system. In practical applications, this system maintains stable denitrification performance above 85.3%, with removal rates of tetracycline antibiotics exceeding 90% [107].

Of the various MBR coupled processes, each configuration presents a distinct set of advantages tailored to specific project goals and constraints [75]: A/O-MBR systems provide the most cost-effective solution for conventional municipal wastewater treatment with moderate nitrogen removal requirements [113]; Multi-stage A/O-MBR configurations offer superior treatment performance and enhanced membrane fouling control, albeit at the expense of higher capital and operational costs; A/A/O-MBR represents the optimal balance for facilities requiring simultaneous nitrogen and phosphorus removal within compact footprints [114]; A/A/O/A-MBR delivers the highest nitrogen removal efficiency among conventional activated sludge-based hybrids, making it suitable for meeting stringent discharge limits; whereas UASB-A/O-MBR demonstrates the best energy sustainability and sludge minimization characteristics, proving particularly advantageous for the treatment of high-strength industrial wastewater [115]. This comprehensive comparison enables engineers and plant designers to select the most appropriate MBR coupling strategy based on specific treatment objectives, available resources, and local economic constraints.

### 4.2. Enhanced Denitrification via Coupled Pretreatment Processes

MBR systems achieve enhanced denitrification through integration with various pretreatment techniques, as summarized in Table 4. Typical strategies include the addition of flocculants [116], ozone (O_3_) oxidation [117], powdered activated carbon (PAC) dosing [26,118], and electro-activation modules [119]. Coagulation pretreatment prior to MBR promotes flocculation and settling of pollutants, significantly reducing organic and nitrogen loads on the subsequent biological process. This reduction creates favorable conditions for the enrichment of nitrifying bacteria, thereby improving denitrification performance [119]. This is a low-cost and easily scalable strategy, but it increases sludge production and associated disposal costs.

In the O_3_-MBR process applied to landfill leachate treatment, pre-ozonation selectively oxidizes and cleaves aromatic rings in recalcitrant organic compounds, converting high-molecular-weight humic substances into more biodegradable fractions such as fulvic acid and humic acid [120,124]. These small-molecule organic acids serve as high-quality carbon sources for microbial metabolism, stimulating the activity of nitrifying bacteria (e.g., *Nitrospira* spp.) and denitrifying bacteria (e.g., *Thauera* spp.) in the MBR. This approach not only sustains efficient removal of ammonia and total nitrogen but also mitigates membrane fouling by oxidizing foulant precursors, thereby reducing operational costs [121]. However, the high energy consumption for ozone generation and the potential formation of bromate by-products are key economic and environmental concerns, requiring careful process control for sustainable application.

In the PAC-MBR system, PAC serves a dual role: its porous structure adsorbs dissolved organic nitrogen and refractory pollutants, while also acting as a biofilm carrier that supports microbial growth and reproduction [125,126]. This enhances the overall microbial degradation capacity. Furthermore, PAC promotes the formation of granular sludge, creating distinct dissolved oxygen microenvironments on the surface and inside sludge particles. These conditions support alternating nitrification and denitrification, thereby improving nitrogen removal efficiency [127]. The operational costs are primarily associated with PAC replenishment, but this can be offset by reduced membrane cleaning frequency and improved process stability. Its scalability is well-proven in both municipal and industrial applications.

Incorporating electrochemical methods into MBR generates highly reactive radicals that accelerate ammonia oxidation [122]. As shown in Figure 5c,d, the electroconductive moving bed membrane bioreactor developed by Udomkittayachai et al. achieved enhanced nitrogen removal (TN removal of 88.8%) while effectively mitigating membrane fouling and lowering its energy requirements through the addition of conductive media and intermittent electrical exposure [128]. The electron transfer mechanisms in ECMB-MBR primarily involve two pathways: (1) direct electron transfer from electrodes to microorganisms or between microbial species through conductive pili or cytochromes, and (2) indirect electron transfer via mediators such as hydrogen or formate generated in situ at the cathode. These mechanisms facilitate the oxidation of ammonia and the reduction in nitrate/nitrite, enhancing the overall nitrogen removal efficiency. Regarding cost implications, while the electrical system increases energy consumption, this can be balanced by multiple benefits: significant reduction in membrane cleaning chemicals and downtime, decreased sludge production (up to 20–30% reduction), and enhanced treatment capacity allowing for more compact reactor designs. The net effect can be a reduction in overall operating costs by 15–25% despite the additional energy input, making it economically viable for high-strength wastewater treatment where conventional processes face challenges.

Simultaneously, the resulting micro-electric fields modify sludge properties and promote the proliferation of functional microbial communities [129]. As a result, the nitrogen removal rate (NRR) increases by up to 30%, with stable performance improvements observed in the treatment of high-ammonia wastewater [130]. Despite higher capital and operational energy costs for the electrical system, the ECMB-MBR can achieve significant savings through reduced sludge production and membrane fouling control, showing promise for niche, high-strength applications, though full-scale scalability needs further demonstration.

### 4.3. Enhancing Denitrification Through Sludge Property Regulation

For MBR systems, the regulation of sludge properties represents an effective strategy to enhance nitrogen removal efficiency while enabling broad-spectrum pollutant elimination, as summarized in Table 5. Fillers modify sludge structure, their porous architecture helps optimize floc morphology and reduce sludge loss [131]. Moreover, their customizable nature allows for functional modifications that further improve sludge settleability and microbial colonization capacity [132]. By incorporating media additions [48] and modification [133,134], MBR systems can thus enhance sludge characteristics and thereby improve denitrification performance. Compared to conventional MBR, this approach improves biological treatment efficiency and enriches specific microorganisms through biofilm formation on filler, addressing long start-up and operational instability [135].

In the suspended media-MBR process, the addition of media improves sludge dispersion, which in turn enhances microbial activity and metabolic capacity, leading to a denitrification efficiency of approximately 72.3% [139]. This is a relatively low-cost modification with minimal energy input, offering easy integration into existing tanks and good scalability for capacity expansion. The sponge-coupled degradable carbon source carrier-MBR organically integrates carriers with biodegradable carbon sources to establish targeted denitrification pathways, supplying functional microorganisms with a stable carbon source [86]. This configuration increases the activity and metabolic rate of slow-growing functional microbes, raising nitrogen removal to ~94.3% [134]. The mechanism involves sustained internal carbon release, eliminating the need for complex external carbon dosing. While carrier cost is a factor, the savings from reduced external carbon source addition and improved process stability can be significant, making it scalable for low C/N wastewater treatment [136].

The sandwich-type carrier-MBR creates a favorable microenvironment for anammox bacteria, enabling their effective enrichment and resulting in a denitrification capacity of approximately 70.6% [137]. Its structured design mechanically supports anammox biofilms, protecting them from washout. The primary cost involves the specialized carrier, but it drastically reduces start-up time for anammox processes, offering a potentially cost-effective solution for mainstream anammox application after further carrier cost optimization.

**Figure 6:**
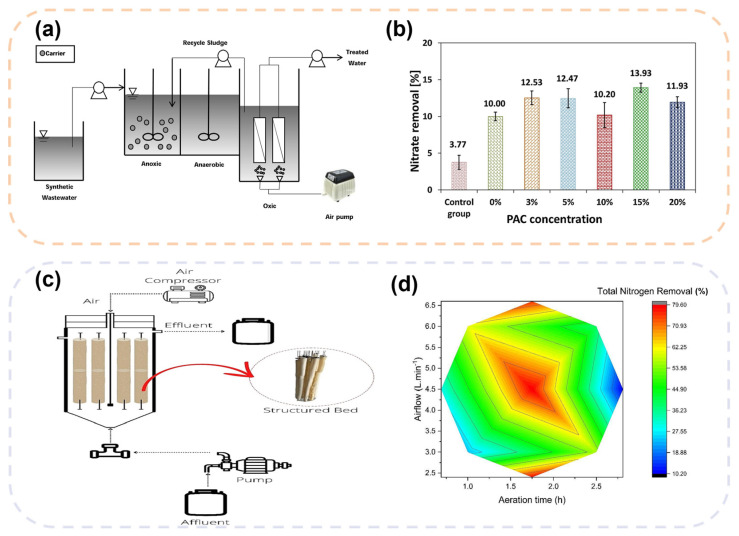
Schematic diagrams and performance analysis of biofilm mechanisms and carrier-based denitrification processes. (**a**) Schematic diagram of the MBR process for enhanced denitrification coupling sulfur-based carriers with PAC; (**b**) Comparison of nitrate removal efficiency under different PAC concentrations in sulfur-based carrier MBR [26]; (**c**) Schematic diagram of the structured bed reactor (SBRIA) system for biofilm-based nitrogen removal; (**d**) Response surface plot of total nitrogen removal efficiency as a function of aeration time and aeration intensity in SBRIA [143].

In addition, growing research attention is being directed toward sulfur-based carrier-MBR systems. As shown in Figure 6a,b, Woo et al. [128]. developed a sulfur-based carrier incorporating PAC. When applied in an MBR system, the total nitrogen removal efficiency increased by approximately 40% compared to the control without the carrier, reaching 72.7%, without compromising the removal of organic matter. The carrier not only provided attachment sites and electron donors for sulfur-oxidizing autotrophic denitrifying bacteria, but the addition of PAC further enhanced the reaction rate, demonstrating the feasibility of using sulfur-based carriers to enhance denitrification in MBRs without the need for external organic carbon sources [26]. This approach leverages autotrophic denitrification, minimizing sludge production and operational costs associated with carbon sourcing. However, the potential accumulation of sulfate in the effluent and the long-term stability of the sulfur carrier are key considerations for its economic viability and large-scale applicability [140].

### 4.4. Enhanced Denitrification Through Functional Biofilm Formation

Biofilms are structured microbial communities encased in a self-produced extracellular polymeric matrix and attached to living or inert surfaces [79,141]. These assemblages exhibit complex internal heterogeneity, forming diverse microenvironments that enable synergistic interactions among microorganisms with complementary metabolic functions. In MBR systems, biofilm architecture on membrane surfaces depends on bacterial community composition and environmental conditions like DO and temperature [142]. While the taxonomic profile defines the intrinsic metabolic potential of functional populations, environmental factors modulate the actual expression of this potential by regulating microbial activity. As a result, variations in microbial community structure ultimately determine the system’s nitrogen removal efficiency [130]. As shown in Figure 6c,d, Paniguel et al. [143] established a biofilm system in an intermittently aerated structured bed reactor dominated by the *phyla Proteobacteria* and *Planctomycetota*. The former is a widespread denitrifying phylum, with classes such as *β-Proteobacteria* and *γ-Proteobacteria* representing some of the most extensively studied denitrifying bacterial groups [144], capable of driving denitrification via a suite of specialized enzymes. The latter phylum is well-known for anammox, genera such as *Candidatus Brocadia* directly convert ammonia and nitrite into nitrogen gas [138]. The coexistence of denitrification and anammox in this system enables the nitrogen removal efficiency to reach 79.6%. Such biofilm systems often exhibit greater resilience to toxic shocks and load variations compared to suspended growth systems. The primary costs are associated with the carrier media, but these are often offset by reduced reactor volume requirements and lower sludge production, favoring their scalability for compact plant designs.

In a related study, Zhu et al. [145] achieved PN/AMX by applying an ultra-low dissolved oxygen strategy in the aerobic zone. This approach effectively suppressed NOB while promoting the enrichment of anammox bacteria, increasing the relative abundance of *Candidatus Brocadia* from 0.26% to 1.78% and achieving a total nitrogen removal rate of 93.3%. This strategy significantly reduces aeration energy costs, a major operational expense. The challenge for scalability lies in the precise and reliable control of DO at ultra-low levels across large-scale reactors. Further supporting these findings, studies on anammox-MBR (AX-MBR) coupled processes have shown that anammox bacteria can account for up to 65% of nitrogen removal, underscoring the critical role of targeted microbial enrichment in enhancing MBR denitrification performance [146]. The main economic benefit of AX-MBR is the drastic reduction in aeration energy and external carbon demand. While the long start-up time remains a hurdle for widespread adoption, the operational cost savings present a strong driver for its implementation in energy-neutral or positive wastewater treatment schemes [147].

## 5. Conclusions and Outlook

The MBR process exhibits excellent denitrification performance, effectively ensuring treatment efficacy and compliance with discharge standards. Current research is primarily directed toward enhancing nitrogen removal through strategies such as process coupling, pretreatment intensification, regulation of sludge properties, and construction of functional biofilms. However, to transcend conventional treatment paradigms and align with sustainability goals, future research should prioritize bridging critical knowledge gaps and embracing emerging trends such as circular economy integration and carbon–neutral operation. Key directions include (1) advancing resource recovery by coupling MBR with nitrogen and phosphorus recycling processes and facilitating water reuse, rather than focusing solely on removal; (2) achieving carbon-neutral operation through energy-efficient configurations, utilization of anaerobic processes for energy recovery, and direct mitigation of N_2_O emissions; (3) enabling precise microbial community management via multi-omics insights to regulate metabolic pathways and interspecies interactions for stable and efficient nitrogen removal; and (4) integrating smart control systems and advanced oxidation processes to optimize operational parameters, mitigate membrane fouling, and enhance resilience to emerging contaminants. By addressing these priorities, MBR technology can evolve from a treatment-centric solution to a sustainable, resource-oriented platform that supports the transformation of wastewater treatment plants into energy-positive and resource-recovering facilities.

## Figures and Tables

**Table 1:** Key operational factors influencing denitrification performance in MBR systems.

Factor	Optimal Range	Primary Influence on Microorganisms and Process	Reference
pH	7.0–8.0	Optimizes enzyme activity for nitrification and denitrification; influences microbial surface charge and floc stability. Critical for Anammox and DAMO processes.	[57,58,60]
Temperature	30–35 °C (for PN/AMX)	Increases microbial metabolic rates; can selectively favor AOB over NOB to promote nitrite accumulation for advanced pathways.	[61]
DO	0.5–1.5 mg/L (for SND)	Balances nitrification (requiring O_2_) with denitrification (requiring anoxia). Low DO enables simultaneous nitrification–denitrification within flocs.	[62]
C/N Ratio	>5 (Heterotrophic)	Sufficient organic carbon is required as an electron donor for heterotrophic denitrification. Low C/N necessitates autotrophic pathways.	[64]
HRT	Reactor-specific (e.g., ~10 h–)	Must be sufficient to allow for the growth and metabolism of slow-growing microorganisms (e.g., nitrifiers, AnAOB). Too short HRT causes washout.	[65]
SRT	20–50 days	Long SRTs are crucial for enriching slow-growing nitrifying and anaerobic ammonia-oxidizing bacteria, ensuring stable nitrification and advanced nitrogen removal.	[66]

**Table 2:** Types of MBR.

Classification Basis	Type
Combination form of membrane module and bioreactor	EMBR, SMBR, CMBR
Bioreactor	AMBR, AnMBR
Drive configuration	EMBR, SMBR
Membrane module category	tubular, flat-Sheet, hollow-fiber
Membrane material	Organic membrane, Inorganic membrane

**Table 3:** Study on biological nitrogen removal performance of MBR coupled with other processes.

Combined Process	Process Characteristics	Energy Consumption Characteristics (kWh/m^3^)	Sludge Yield (kg MLSS/m^3^ Wastewater)	Total Nitrogen Removal Rate	Reference
A/O-MBR	Suitable for wastewater treatment under most conditions, with a high removal efficiency for organic pollutants.	0.60–0.80	0.20–0.30	≥72.3%	[8,101]
Multi-stage A/O-MBR	Compared to the A/O process, membrane fouling is reduced, process costs are lowered, and treatment efficiency is enhanced.	0.50–0.70	0.16–0.24	≥78.6%	[102,103]
A/A/O-MBR	When integrated with the most prevalent municipal wastewater treatment processes, effluent quality is significantly improved.	0.50–0.65	0.15–0.25	78.6 ± 6.5%	[104,105,106]
A/A/O/A-MBR	The addition of an anoxic zone enhances total nitrogen removal efficiency, offering potential upgrade prospects for the A/A/O-MBR system.	0.45–0.60	0.10–0.20	88.4 ± 7.6%	[24]
UASB-A/O-MBR	It ensures the removal of organic matter and nitrogen while also eliminating pollutants such as antibiotics.	0.40–0.55	0.08–0.18	≥85.3%	[107,108]

**Table 4:** Study on nitrogen removal efficiency of MBR combined pretreatment processes.

Combined Process	Process Characteristics	Energy Consumption Characteristics (kWh/m^3^)	Sludge Yield (kg MLSS/m^3^ Wastewater)	Total Nitrogen Removal Rate	Reference
Coagulation-MBR	Demonstrate excellent removal efficiency for organic pollutants and ammonia nitrogen.	0.55–0.70	0.18–0.25	75% ± 8%	[112]
O_3_-MBR	Effectively remove both organic matter and ammonia nitrogen while also eliminating FA and HA.	0.75–0.90	0.15–0.22	≥78%	[117,120]
PAC-MBR	Microorganism can adsorb within activated carbon, facilitating nitrification and denitrification processes to enhance denitrification efficiency.	0.60–0.75	0.16–0.23	≥79%	[118,121]
Electrochemical-MBR	Use electrochemical properties to assist pollutant removal, exhibiting superior efficacy in eliminating organic contaminants.	0.80–1.00	0.12–0.19	≥85–92%	[122,123]

**Table 5:** Study on nitrogen removal efficiency of MBR with fillers or modified fillers.

Combined Process	Process Characteristics	Energy Consumption Characteristics (kWh/m^3^)	Sludge Yield (kg MLSS/m^3^ Wastewater)	Total Nitrogen Removal Rate	Reference
Suspended filler-MBR	Suspended carriers provide a breeding ground for the enrichment and proliferation of specific microorganisms.	0.60–0.75	0.16–0.23	80 ± 4%	[132]
Sponge Coupled Biodegradable Carbon Source Carrier-MBR	Sponge Coupled Biodegradable Carbon Source Carrier-MBR	0.70–0.90	0.12–0.18	≥94.3%	[136]
Sandwich-type Carrier-MBR	Establishment of a dominant environment for the rapid enrichment of anammox bacteria.	0.55–0.70	0.15–0.22	≥70.6%	[137,138]
Sulfur-based Carrier-MBR	Autotrophic denitrification is driven by using sulfur as the electron donor.	0.60–0.80	0.10–0.15	≥63%	[128]

## Data Availability

No new data were created or analyzed in this study. Data sharing is not applicable to this article.
